# Nano-viscosimetry analysis of the membrane disrupting action of the bee venom peptide melittin

**DOI:** 10.1038/s41598-019-47325-y

**Published:** 2019-07-25

**Authors:** Sara Pandidan, Adam Mechler

**Affiliations:** La Trobe Institute for Molecular Science, La Trobe University, Melbourne, Australia

**Keywords:** Membranes, Peptides, Membrane structure and assembly, Nanometrology

## Abstract

Melittin is one of the most studied α-helical cationic membrane disrupting peptides. It is the main component of bee venom, however it is considered an antimicrobial peptide for its ability to kill bacteria. Melittin is believed to act by opening large toroidal pores in the plasma membrane of the targeted cells/bacteria, although this is questioned by some authors. Little is known, however, about the molecular mechanism leading to this activity. In this study the mechanism of action of melittin was studied by dye leakage and quartz crystal microbalance fingerprinting analysis in biomimetic model membranes. The results revealed the existence of multiple stages in the membrane disrupting action with characteristic differences between different membrane types. In bacterial-mimetic (charged) lipid mixtures the viscoelastic fingerprints suggest a surface-acting mechanism, whereas in mammalian-mimetic (neutral) membranes melittin appears to penetrate the bilayer already at low concentrations. In domain-forming mixed membranes melittin shows a preference for the domain containing predominantly zwitterionic lipids. The results confirm membrane poration but are inconsistent with the insertion-to-toroidal pore pathway. Therefore hypotheses of the two membrane disrupting pathways were developed, describing the membrane disruption as either surface tension modulation leading to toroidal pore formation, or linear aggregation leading to fissure formation in the membrane.

## Introduction

The emergence of bacterial resistance to conventional antibiotics is one of the most significant international health problems of our time that requires an urgent solution^1^. A potential answer to this near-crisis situation is using antimicrobial peptides (AMPs)^2^. AMPs represent one of the oldest, highly effective forms of antimicrobial defence in all complex organisms, including plants, insects, amphibians and even mammals, typically released in response to stress^3^ and hazardous situations, such as facing the risk of a predator’s attack^4^. Many AMPs can kill both Gram-positive and Gram-negative bacteria, while some of them have anticancer and antiviral activities as well^5^. Particularly interesting are cationic α-helical AMPs that kill bacteria by disrupting their plasma membrane, offering a specific class of potential therapeutic agents that are complementary to existing antibiotics, and which bacteria may not be able to develop resistance toward^6,7^.

To identify sources of bioactive natural products traditional medicine is often a good guidance. Bee venom therapy is one of the methods in traditional medicine, used to treat different ailments^8,9^. Bee venom is a complex mixture of peptides and enzymes, and the peptide melittin is the main component that constitutes 50% of the dry weight of European honey bee (Apis mellifera) venom^10^. Melittin is essentially a broad spectrum cell lysing agent^11^ that is nevertheless generally known as an antimicrobial peptide in the literature^12^ as it exerted strong antibacterial activity on 51 strains of both Gram-positive and Gram-negative bacteria, as well as rapidly growing mycobacteria^13^. However, melittin’s potent antimicrobial activity is hitherto inseparable from its haemolytic action^12^. Hence, melittin is a case study of membrane disrupting peptides; its potential for drug development can only be realized once its mechanism of action is fully understood, and its sequence is engineered in a way that it can selectively target bacteria.

Melittin is α-helical, cationic and amphiphilic peptide that consists of 26 amino acids (NH_2_-GIGAVLKVLTTGLPALISWIKRKRQQ-CONH_2_). Its C-terminal domain contains positively charged residues and hence is hydrophilic, while the N-terminal region is mostly hydrophobic^10,14^. Despite the high proportion of hydrophobic side chains in its sequence, melittin is highly soluble in water, and moderately soluble in methanol^15^. The structure of melittin was determined by X-ray diffraction^16^ and NMR spectroscopy in methanol solution^17^. The folding of melittin consists of two α-helical domains (residues 1–10 and 13–26) intersecting at an angle of 120°, due to a proline “kink” to form a bent structure; in the longer helix the hydrophilic and hydrophobic residues are aligned, facing in opposite directions^15,18^. According to CD measurements melittin remains unfolded in aqueous solution, but becomes 68% α-helical when interacting with a DMPC bilayer; notably, the helicity is only 32% when cholesterol is mixed to the phospholipid^19^, suggesting that mammalian cells are protected against lysis and thus melittin exhibits some degree of specificity towards bacterial membranes. That is, however, inconsistent with the reported haemolytic activity^12^ and pore forming activity in membranes of small cholesterol content^20^. However, efforts to develop melittin-based antibiotics have not progressed beyond preclinical phase; like most of AMP derivatives, melittin based drug candidates could not obtain Food & Drug Administration approval^21^.

The key challenge facing drug development from membrane disrupting AMPs is the absence of a clearly defined drug target. The mechanism of action is based solely on a rearrangement of the lipid molecules around the peptides to breach the membrane integrity, such as forming a pore structure of a defined geometry^22–24^. Specifics of this process are challenging to obtain due to the molecular dimensions of the interacting structures^23,25^. Only the end result: the membrane disruption itself can be confirmed with high confidence using dye leakage assays^26,27^, while the details of the pathway leading to membrane breach remain elusive^28–30^. The dynamic nature of the peptide-lipid interaction limits microscopic methods to the capture of large-scale morphology changes, while the location, orientation or even aggregation of the peptides in real time cannot be resolved^31–33^. The same dynamics limit the achievable detail from spectroscopic and surface characterization methods. In the absence of permanent interactions between lipid and peptide moieties, the only sub-molecular resolution data is gleaned by solid-state nuclear magnetic resonance (ss-NMR) measurements that identify disturbances in specific lipid moieties by the interaction with the peptide^34–39^. The generally accepted membrane disruption models were developed with reliance on ss-NMR data^40,41^. However even ss-NMR cannot distinguish multiple, co-existing membrane-bound states of the peptide, and, given that it essentially averages over all present molecules of a given species, it requires very high peptide to lipid ratios to clearly show the effect of the peptide^42^.

Consistently, there is no consensus in the literature about the mechanism of action of melittin, even though it is perhaps the best-known and most studied membrane disrupting peptide^43^. Initially it was believed that melittin induced barrel-stave pores^43,44^. Consecutively a range of studies argued that melittin forms toroidal pores^27,43,45–51^. Molecular dynamics simulations also support the toroidal pore forming mechanism^52^. However a surface plasmon resonance study suggested that melittin can change its mechanism of action as a function of membrane properties, inserting into the membrane core in zwitterionic membranes (incl. 10% cholesterol), and acting in a detergent-like manner in anionic membranes^20^. Dual-colour fluorescence-burst analysis studies using pure DOPC and DOPC:cholesterol 9:1 membranes showed concentration dependent action: at low concentrations, pore formation with the pore size dependent on melittin concentration, whereas at high concentrations and/or on charged membranes melittin triggered liposome fusion and aggregation without specific dye leakage^53^. Melittin-induced pores have never been imaged at a quality that would clearly confirm the toroidal pore hypothesis; indeed an AFM imaging study suggested that melittin is not forming pores at all, rather fissures in the membrane^54^. Thus, there is no clear consensus on the membrane disrupting pathway of melittin; even less is known about the molecular process leading to membrane disruption.

Recent improvement in multimodal surface characterizing methods such as quartz crystal microbalance “with dissipation” (QCM) opened new ways to glean further insights into AMP-membrane interactions. QCM measures mass with nanogram sensitivity simultaneously with recording any changes in the viscosity of the sample^55–58^. This can be used to produce viscoelastic fingerprints of the membrane interaction, revealing distinct stages of the process^31,59–64^. It should be noted that these stages differ in their viscoelastic character; to assign them to actual molecular processes either further information is needed about the peptide-membrane interaction, or at least a model based on the physical chemistry of peptide-membrane inetractions.

In this work the mechanism of melittin interaction with different lipid mixtures was studied using QCM as the main method, while dye leakage measurements were used to benchmark the peptide activity. Based on viscoelastic fingerprint analysis to identify stages of the membrane interaction, two new mechanistic pathways were proposed for these model membranes.

## Materials and Methods

### Buffer preparation

Potassium dihydrogen phosphate (KH_2_PO_4_) was purchased from Sigma Aldrich (Castle Hill, NSW, Australia) and Fluka branded potassium hydrogen phosphate (K_2_HPO_4_) was purchased from Honeywell (Shanghai, China). Sodium chloride (NaCl) was purchased from Chem-Supply Pty Ltd, Gillman SA, Australia. The phosphate buffered saline (PBS) assay buffer contained 20 mM phosphate and 100 mM NaCl at pH 7.2. Deionized water of 18.2 MΩcm resistivity (Ultrapure, Sartorius AG, Germany) was used for all solutions.

### Vesicle preparation

1,2-dimyristoyl-sn-glycero-3-phosphocholine(DMPC), 1,2-dioleoyl-sn-glycero-3-phosphocholine (DOPC), sodium salt of 1,2-dimyristoyl-sn-glycero-3-phosphoglycerol (DMPG), and cholesterol were purchased from Avanti Polar Lipids (Alabaster, AL, USA). Chloroform (ACS Reagent, 99.8%) and methanol (>99.9%, spectrophotometric grade) were purchased from Sigma-Aldrich (Castle Hill, NSW, Australia).

Lyophilized lipids were dissolved in chloroform; in case of DMPG 3% methanol was also added to increase solubility. Lipid solutions were mixed in desired ratios in a clean test tube. As described before, the solvent was evaporated with a gentle flow of nitrogen gas upon continuous moderate vortexing to obtain a uniform layer. Afterward, the tubes were stored in a desiccator. Before use the lipid aliquots were hydrated in 1 ml PBS in a 37 °C incubator for 30 minutes, followed by one minute vortexing and brief sonication. This method yields a broad distribution of mostly unilamellar liposomes; the membrane deposition process is optimized for using this precursor^59^. Lipid suspensions were used immediately.

As mammalian-mimetic membrane model, neat DMPC, DOPC, DMPC:cholesterol (9:1) and DMPC/chol (8:2) mixtures were used; as bacteria-mimetic model membranes DMPC:DMPG (4:1) and DMPC:DMPG (3:2) mixtures were used. While this approach reduces the complexity of natural membrane, it allows for studying the effect of each membrane constituent, in line with our previous work and also a common practice in the literature^64–68^.

### Dye leakage assay

Vesicles were loaded with 5(6)-Caboxyfluoresceine purchased from Sigma-Aldrich (Castle Hill, Australia). The lipid samples were suspended in PBS buffer containing 0.02 M CF dye solution and incubated for 30 minutes, following with one-minute vortex and one-minute sonication. Excess CF was removed from the medium by dialysis using SnakeSkin Dialysis Tubing 10000 MWCO, replacing the buffer solution 3 times until a clear solution was obtained. All experiments were performed using a SpectraMax M5 Multi-Mode Microplate Reader. Intensities were recorded every 60 s for 45 min after adding 1, 3, 5, 7 and 10 µM melittin at 25 °C with excitation and emission wavelengths of 480 nm and 517 nm, respectively. The measurements were repeated at least three times for each membrane type and peptide concentration; the results showed high qualitative and quantitative agreement.

### Quartz crystal microbalance

All experiments were conducted with a Q-SENSE E4 system (Q-SENSE, Sweden) using AT-cut gold-coated quartz chips with a fundamental resonance frequency of 5 MHz. QCM chips were cleaned and oxidised with a base piranha solution consisting of 1:1:3 of hydrogen peroxide (aqueous solution, 30%) purchased from ChemSupply Pty Ltd, Australia, Gillman, SA; ammonia solution (28–30%) purchased from Merck, Darmstadt Germany; and deionized water, at 70 °C for 20 minutes.

The cleaned chips were rinsed with ultra-pure water and dried under a gentle stream of N_2_. The chips were modified in a solution of 2% 3-mercaptopropionic acid (MPA) (HPLC Grade, >99%, Fluka branded product from Sigma Aldrich, Castle Hill, NSW, Australia) in propan-2-ol overnight. Next, the chips were soaked in propan-2-ol to remove any excess thiol from the surface. After subsequent drying the chips were placed in deionized water for a few hours to hydrate the MPA self-assembled monolayer.

QCM is primarily used for measuring mass changes, such as peptide binding to a specific membrane. The second information channel, energy dissipation, is often neglected as most model membrane platforms restrain dissipative processes in supported membranes. By using a partially suspended membrane system, the dissipation signal becomes a useful measure of viscoelastic changes in the membrane properties and thus it can be also used to glean further insights into the peptide-membrane interaction^69,70^.

### Lipid deposition and fingerprinting assays

After installing the MPA modified chips in to the QCM chambers, baselines were recorded first in deionized water and in PBS for reference and also to confirm the stability of the sensor signals. Next, the lipid suspensions were injected into the chambers. Deposition was monitored in real time and, when a stable baseline was reached, mild osmotic stress (20 mM phosphate buffer solution without salt) was also used to clean the membrane surface of liposome residues. Assay buffer was then returned into the chamber and melittin (GL Biochem Ltd., Shanghai) was introduced in desired concentrations. All experiments were repeated at least three times at 19 °C and at a flow rate of 50 µL/min. The frequency and dissipation changes were only plotted for four eigenmodes (3^rd^, 5^th^, 7^th^ and 9^th^; 15–45 MHz) as these provide the most reliable data on lipid membranes^59^.

In order to analyse the peptide mechanism of action, the QCM results were converted into f-D curves. These viscoelastic fingerprints show different stages of the membrane disruption process as distinct trendlines, and may be analysed to reveal the molecular process of peptide-membrane interaction as described before.

### Dynamic light scattering (DLS) assay

DLS measurements were performed using a Malvern Zetasizer Nano instrument (Malvern Instruments, Ltd., Malvern Worcestershire, UK) and disposable micro cuvettes (ZEN0040). Measurements were done at 25 °C on 2 µM DMPC before and after adding 10 µM melittin. In the latter case the samples were centrifuged at 3000 rpm for 10 min and the supernatant was passed through a 200 nm pore size syringe filter to remove any intact liposomes that may obscure the small populations.

## Results and Discussion

### Dye leakage assays

**Figure 1:**
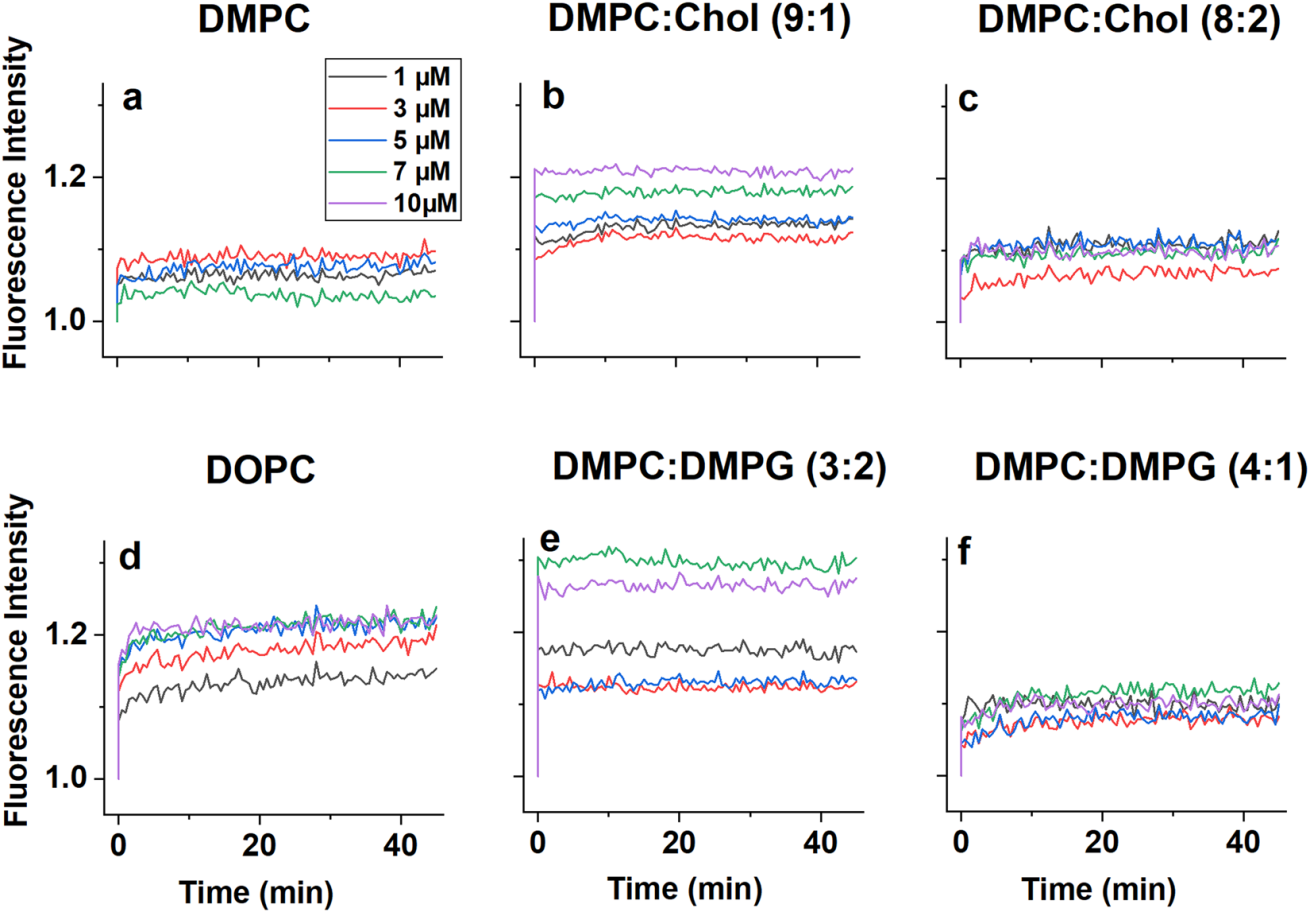
Results of dye leakage experiments. Normalized intensity of dye release shown as a function of time for a range of membrane mixtures and peptide concentrations as indicated. (**a**) DMPC; (**b**) DMPC:cholesterol (9:1); (**c**) DMPC:Cholesterol (8:2); (**d**) DOPC; (**e**) DMPC:DMPG (3:2); (**f**) DMPC:DMPG (4:1). The fluorescence intensity of dye loaded liposomes before the addition of the peptide was set as unity.

Figure 1 shows the dye leakage results. In this work, the dye concentration was only slightly higher than the lower limit of self-quenching concentration to avoid interference from osmotic effects. Consistently, the intensity increases are moderate.

Exposing DMPC liposomes to melittin causes immediate, thresholdless and concentration-independent intensity increase to 4–8% (Fig. 1a). The result is nearly identical if DMPC is mixed with 20% cholesterol (Fig. 1c). However, in case of 10% cholesterol content the dye leakage is both higher and clearly concentration dependent, starting from ~12% at 1 μM melittin and reaching 20% at 10 μM concentration (Fig. 1b). The dye leakage trend on the unsaturated DOPC membrane (Fig. 1d) was near identical to the DMPC:cholesterol (9:1).

In case of the bacteria-mimetic membranes, the kinetics of the dye leakage appear different. For DMPC:DMPG (4:1) the intensity reaches a maximum of ~ 10% increase in approximately 20 minutes, in a concentration-independent manner. However when increasing the DMPG content to 40%, the dye leakage is again immediate, with an apparent decreasing trend from 1 μM to 5 μM melittin concentration, then a step increase in intensity from 13% to 30%. All the presented results are highly reproducible.

The variations confirm that, while melittin is an indiscriminate cell killer, it exhibits higher activity against the bacteria-mimetic DMPC:DMPG (3:2) membrane. The results also suggest that its action is affected by the structure of the membrane core, that is, whether the lipid molecules are saturated or unsaturated, and the presence and amount of cholesterol^64^.

### QCM assays

#### Mammalian-mimetic membranes

**Figure 2:**
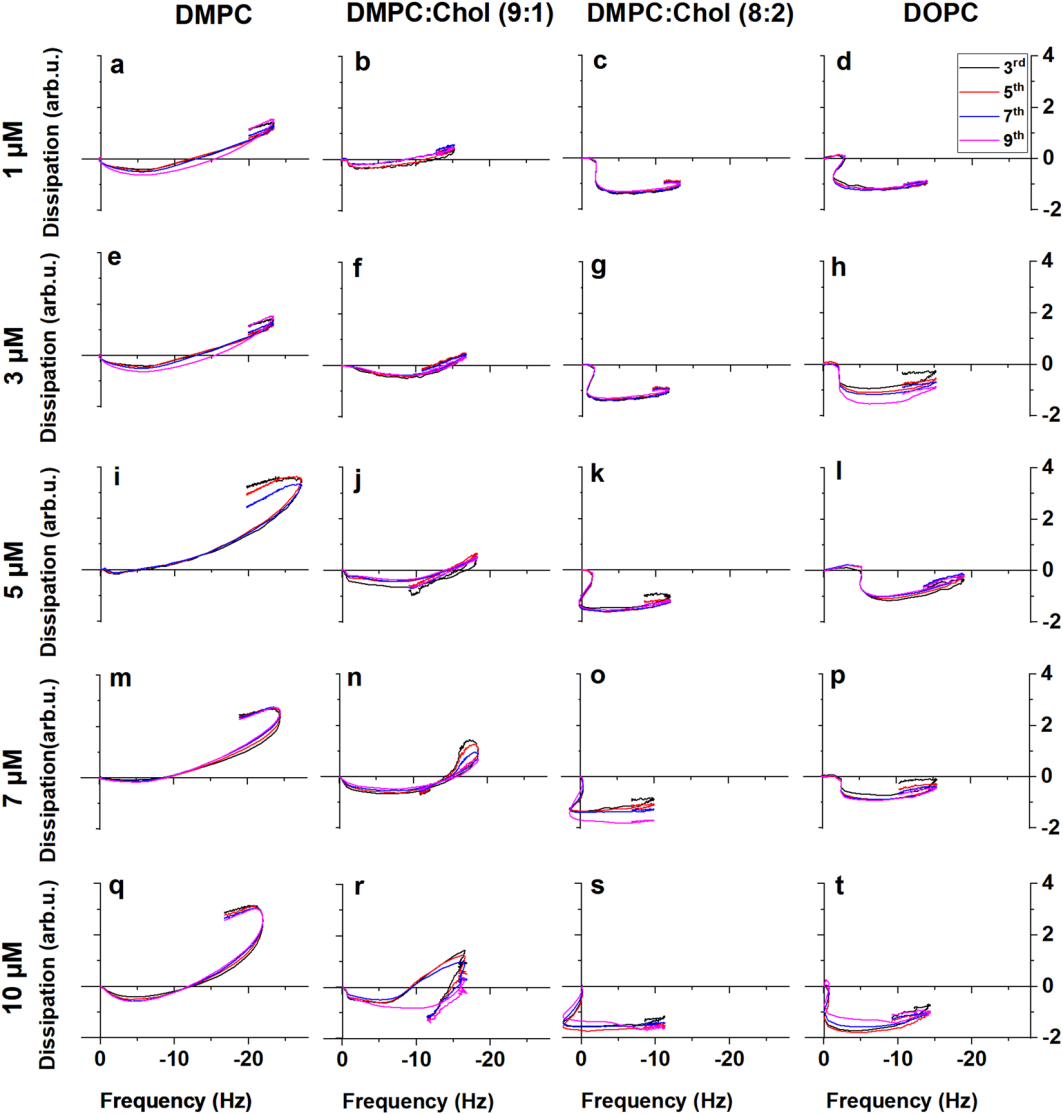
Viscoelastic fingerprints of interactions of melittin at varied concentrations with neutral membranes as follows. (**a**–**d**) 1 μM melittin, (**a**) DMPC, (**b**) DMPC:cholesterol (9:1), (**c**) DMPC:cholesterol (8:2), (**d**) DOPC. (**e–h**) 3 μM melittin, (**e**) DMPC, (**f**) DMPC:cholesterol (9:1), (**g**) DMPC:cholesterol (8:2), (**h**) DOPC. (**i–l**) 5 μM melittin, (**i**) DMPC, (**j**) DMPC:cholesterol (9:1), (**k**) DMPC:cholesterol (8:2), (**l**) DOPC. (**m**–**p**) 7 μM melittin, m) DMPC, (**n**) DMPC:cholesterol (9:1), (**o**) DMPC:cholesterol (8:2), (**p**) DOPC. (**q**–**t**) 10 μM melittin, (**q**) DMPC, (**r**) DMPC:cholesterol (9:1), (**s**) DMPC:cholesterol (8:2), (**t**) DOPC.

As shown in Fig. 2, in the case of neat zwitterionic DMPC membrane, the f-D fingerprints only change in proportion as a function of melittin concentration, but not in their shape. The first noticeable feature is that the first trendline is [-f, -D], unlike the typical [-f,+D] that is common in previously studied peptides^61,64,70–72^. The [-f, -D] trendline suggests that, parallel with mass gain due to the peptides binding to the membrane, an immediate structural change takes place, one that reduces the freedom of the membrane to dissipate energy. This is consistent with immediate peptide penetration into the membrane, altering the lipid packing order and inhibiting out of plane motion, which is the main pathway of energy dissipation in suspended membranes. The fingerprints at different eigenmodes almost perfectly overlap, suggesting that the changes in the membrane structure are more elastic than viscous in character, and may indicate transmembrane insertion of the peptides^31^. This stage reaches approximately the same values at all concentrations: Δf = −4–5 Hz and ΔD = −0.2–0.5 arb. u., suggesting that the occupancy of the membrane inserted monomeric state reaches maximum at these values. Importantly that is achieved already at micromolar bulk peptide concentrations. Thus, melittin appears to preferentially absorb into the membrane from the solution phase in spite of its well-known high aqueous solubility.

Mass uptake (−f) continues in the second stage; however energy dissipation increases after the minimum reached at the end of stage 1, suggesting weakening in-plane interactions that re-introduce freedom of out-of-plane motion and hence dissipative processes. This is consistent with the formation of structures that break the continuity of the membrane, offering several pathways for energy dissipation. Membrane breach takes place parallel with further peptide insertion into the membrane, which contributes to further mass increase (−f). Finally, a slight [+f, −D] mass loss trendline suggests that some fragments of the membrane break free from the surface, likely as a result of expansion of the membrane and “flaking” at the edges (stage 3).

In the presence of 10% cholesterol at low melittin concentrations the frequency trend of the f-D curve is similar to neat DMPC, however the dissipation change is significantly less (Fig. 2b). At 7 μM melittin concentration the fingerprint changes, with a loop continuing towards [+f, -D]; this becomes a sharp turn at 10 μM concentration.

Increasing the amount of cholesterol to 20% in the membrane lead to a substantial change in the shape of the f-D curves. The fingerprints reveal three distinct stages. The brief first stage shows a [-f, 0] trend. A second stage appears as a sudden dissipation drop at ~−2 Hz. At low peptide concentrations this drop takes place without any frequency change, however it acquires an increasingly +f direction as the peptide concentration is increased. This trend is similar to the sudden -D turn observed at 10 μM melittin concentration for DMPC:cholesterol (9:1).

The cause of this sudden drop in dissipation is unclear. In light of the dye leakage results, such a major structural change in the membrane has to be related to membrane disruption; yet if it is pore formation it is very different from the neat DMPC membrane. This is followed by a stage of significant negative frequency change with very small changes in dissipation. In previous works on surface acting peptides it was identified as the collapse of the partially suspended membrane after disruption with continuing adsorption of more peptide^64,70^. Considering the dye leakage results it is feasible to assume a similar process. There is again a small amount of material removal at the end of the process likely due to desorption of peptides upon buffer wash.

The difference between the two cholesterol-containing membranes may be understood based on their structural characteristics. It is known that adding cholesterol to DMPC yields the formation of two domains: DMPC-rich and cholesterol-rich. The DMPC-rich domain shrinks progressively with increasing cholesterol content, disappearing at ~20% cholesterol^73^. It was reported before that the existence of domains of different cholesterol content can influence peptide-membrane interaction, as cholesterol may inhibit penetration and fragmentation of the membrane by some AMPs^74,75^. The QCM data suggest that at 10% cholesterol concentration melittin first interacts with the DMPC-rich domain, hence the similar trend; the lower dissipation is the result of the rigidifying effect of the cholesterol-rich domains. At high melittin concentration the peptides start to interact with the cholesterol-rich domain, resulting in the sudden -D trend, but do not proceed to the [-f, 0] trendline likely due to the different viscoelastic character of the domain separated nature of the membrane. Thus, the results for 10% cholesterol reflect the composite effect of the two distinct membrane domains.

To separate cholesterol effects from the lipid packing in the membrane core, experiments were also performed with unsaturated DOPC membrane and the results are shown in Fig. 2d. The fingerprints show a high degree of similarity to the DMPC:cholesterol (8:2) membrane, that is, to the case when cholesterol distributes evenly in the membrane without forming any domains. This suggests that the peptide insertion is directly affected by the packing and/or viscosity of the membrane core, not by a specific interaction with cholesterol.

#### Bacteria - mimetic membranes

**Figure 3:**
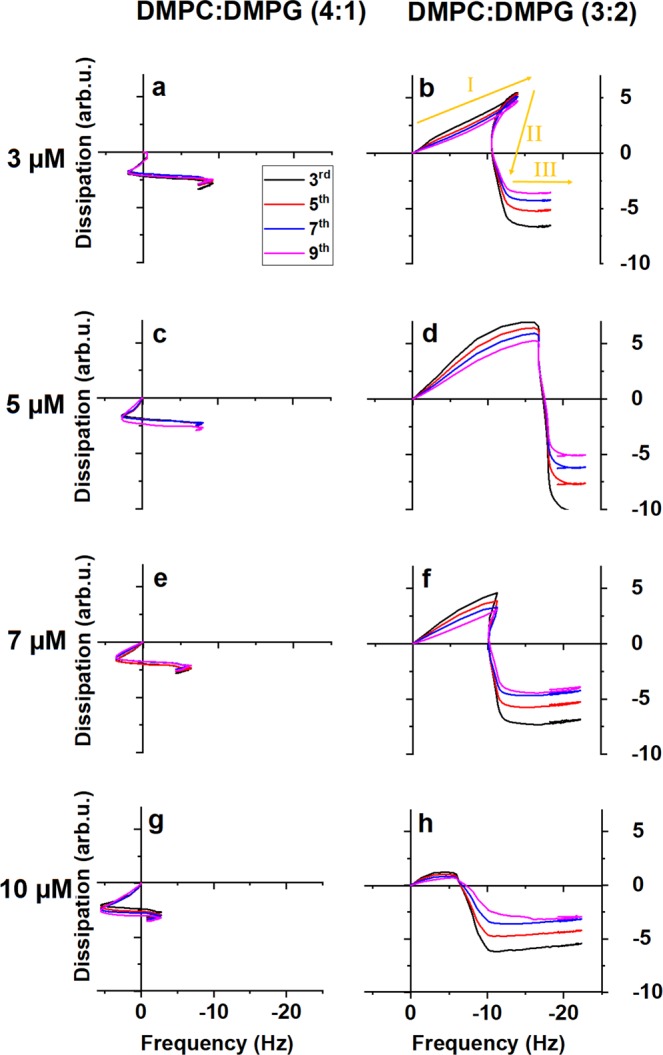
Viscoelastic fingerprints of melittin interactions with anionic membranes at different concentrations as follows. 3 μM melittin: (**a**) DMPC/DMPG (4:1), (**b**) DMPC/DMPG (3:2). 5 μM melittin: (**c**) DMPC/DMPG (4:1), (**d**) DMPC/DMPG (3:2). 7 μM melittin: (**e**) DMPC/DMPG (4:1), (**f**) DMPC/DMPG (3:2). 10 μM melittin: (**g**) DMPC/DMPG (4:1), (**h**) DMPC/DMPG (3:2). The arrows in panel (**b**) indicate stages of the interaction with Roman numerals.

Figure 3 shows f-D curves of melittin interaction with the bacteria-mimetic DMPC:DMPG (4:1) mixture. There are three distinct stages. In all cases, the fingerprints start with a [+f,-D] trend, that abruptly switches into a [–f, 0] trendline with a final stage where this trend is inverted (upon rinsing with assay buffer). The maximum positive frequency change in the first trend is proportional to peptide concentration, whereas the negative frequency change of the second trendline appears to be largely independent of the concentration.

Increasing the anionic (PG) lipid content of the mixture from 20% to 40% yields a much altered fingerprint, as shown in Fig. 2b. The first stage is now a viscoelastic mass increase [-f, +D], consistent with peptides binding to the membrane surface as in case of surface acting peptides; this is followed by a drastic structural change [0, -D], and finally further non-viscous frequency increase [-f, 0] and a very short reverse trend upon washing. The first stage is concentration dependent, however there is a change after 5 μM peptide concentration that has the maximum frequency and dissipation change values: these are reduced again at 7 μM and even more at 10 μM. Importantly, in this lipid mixture the fingerprints show a spreading of the harmonics of the sensor resonance (that overlap in most other cases), which is normally observed in case of surface acting peptides.

The substantial difference in the fingerprints between 20% and 40% anionic lipid content suggests that there is a specific interaction between the PG headgroups and the peptide. DMPC:DMPG (3:2) is the only mixture where the initial trend is similar to the one observed for surface acting peptides; it is also the mixture yielding the strongest dye leakage. Hence it is feasible to assume that here peptides initially stay on the membrane surface, interact with the headgroups and appear as added viscous mass (stage 1). In stage 2 a major structural change commences: as the presence of the peptide increases asymmetric tension, local blistering and/or immediate pore opening leads to a sudden stiffening of the membrane (+f) parallel with a significant reduction of dissipative out-of-plane motion (e.g. undulation) of the membrane (-D). The structural change however allows increased water penetration; this and/or the negative effect of the growing pores on overall membrane elasticity leads to a slight (–f) trend again, especially towards the end of the stage and at higher concentrations. Hence the stage is characterized by curved trendlines. The growing of the pores eventually leads to the membrane collapsing back to the surface (stage 3) as observed before for surface acting peptides^64^.

The difference between the two anionic membranes is analogous to the case of varied cholesterol content as described above. Indeed at a first glance the DMPC:DMPG (4:1) fingerprint is very similar to that of melittin interaction with DMPC:cholesterol (9:1) mixture at 10 μM concentration. It was shown before that DMPC:DMPG (4:1) mixture is also prone to domain separation^59^; it is feasible to assume that the f-D fingerprint reflects a composite effect, similarly to the one described for DMPC:cholesterol (9:1). Therefore in the six studied membranes, we can observe only four clearly different fingerprints: in neat DMPC, neat DOPC, DMPC:cholesterol (8:2) and DMPC:DMPG (3:2). Consistently, the controls of the peptide-membrane interaction are the structure of the membrane core and the presence of phosphatidylglycerol lipids.

### DLS measurements

In order to confirm membrane breakup/dissolution into mixed micelles at high peptide concentrations, DLS measurements have been carried out. DMPC liposomes formed by the method described above typically exhibit two populations: one at ~ 100 nm and a larger, broader size distribution centred at 300–400 nm. When this liposome suspension is exposed to 10 µM melittin, two dominant populations are detected: monomeric peptides at ~1 nm of diameter and a distinct population of uniform sized micelles of approx. 20 nm. A third, small population of intact liposomes of ~ 300 nm diameter was also detected (Fig. S1). These results confirm the formation of mixed micelles/nanodisks as the product of membrane disruption. It should be noted that formation of such mixed micelles was reported before for mutants of magainin2 and melittin^41,75^.

## Mechanism

QCM fingerprints highlighted specific changes in the viscoelastic character of the peptide-membrane interaction, identifying distinct stages of the membrane disrupting process. The identification of these stages is, however, not straightforward, given the complexities of relating the viscoelastic characteristics to ensemble effects of molecular interactions. Therefore further considerations are required. Descriptions of the mechanism of melittin action focus on the final stage, i.e. the membrane poration; thus far not much attention was paid to the pathway that leads to this outcome. In the physicochemical sense the peptide traverses through a number of states characterized by different free energies resulting from the specific physical interactions between the peptide and its environment. Identifying these states helps to assign the viscoelastic trends seen in the QCM experiments.

### Geometric analysis

To establish the physicochemical states of peptide membrane interaction, the membrane-inserted geometry of the peptide is a key clue, of which only indirect data is available in the literature; from the effect on the packing and interactions between the lipid tail groups it is inferred that melittin achieves membrane poration by inserting in a transmembrane manner^15,76–80^. This is based on i) the fact that melittin forms pores without dissolving the membrane, and ii) its length of 26 amino acids, which is considered to be sufficient to form a membrane spanning α-helix. It is important to assess the validity of this argument. It is reported that melittin is unstructured in solution but becomes helical in the membrane environment^81^. In the accepted membrane disruption models the helical form is the prerequisite of membrane penetration and consecutive disruption, since helicity creates the longitudinal amphiphilic character of the N-terminal domain of the peptide. Melittin structure also exhibits a characteristic “kink” due to a proline residue. It is argued that the proline residue plays a key role in both the antimicrobial activity and the (undesired) cytotoxicity of the peptide^82^. Adjacent to the proline the C-terminal domain has a single apolar loop with a tryptophan residue, followed by a “tail” of charged residues.

In a membrane inserted monomeric state the mostly apolar regions of the peptide (residues 1–19) would reach energy minimum in the membrane core. It can be approximated that this helical segment is ~ 2.8 nm long^83^. If this helix enters the hydrophobic membrane core, the tryptophan residue is located close to the ester moieties; by analogy to the role of aromatic rings in other peptides^84–89^ it is feasible to assume that the tryptophan side chain is associated with the esters. This anchoring effect and the following highly charged C-terminal segment that is likely associated to the phosphate moieties set a firm upper limit to membrane penetration at 2.8 nm. However it is unlikely that this helix is inserted normal to the membrane plane, given the polar residues occupying one side that likely retain some hydration and/or association to the polar headgroup area. If the helix is tilted to e.g. 45°, the insertion depth is reduced to ~ 2 nm. Hence the question is whether such a penetration depth is able to destabilize two membrane leaflets.

Neutron diffraction methods established that the hydrophobic thickness of the membrane core is 2.6–3.05 nm^90–92^. At the lower limit, a vertically inserted peptide could bridge the two leaflets, however at the upper limit a tilted peptide may only interact with a single leaflet. A temperature-resolved Raman spectroscopy study using DMPC established that, even at a 14:1 lipid:peptide ratio, melittin interacts only with 5–7 lipid molecules, while simple geometrical considerations would suggest 14 nearest neighbours if the peptide penetrates both leaflets vertically^93^. Based on this it is highly possible that there are circumstances where melittin molecules interact only with a single membrane leaflet. Potentially the tilt of the peptide depends on the strength of the interactions of the polar moieties of the N-terminal helix with the membrane headgroups, explaining the differences between the mammalian and bacterial-mimetic membranes. However, even if melittin does not insert in a transmembrane manner, it is well established that it disrupts most natural and biomimetic membranes. This suggests that melittin can trigger distortion of the membrane structure even if it is not in a transmembrane orientation. In the followings two hypotheses are presented to describe such a mechanism.

### Fissure hypothesis

**Figure 4:**
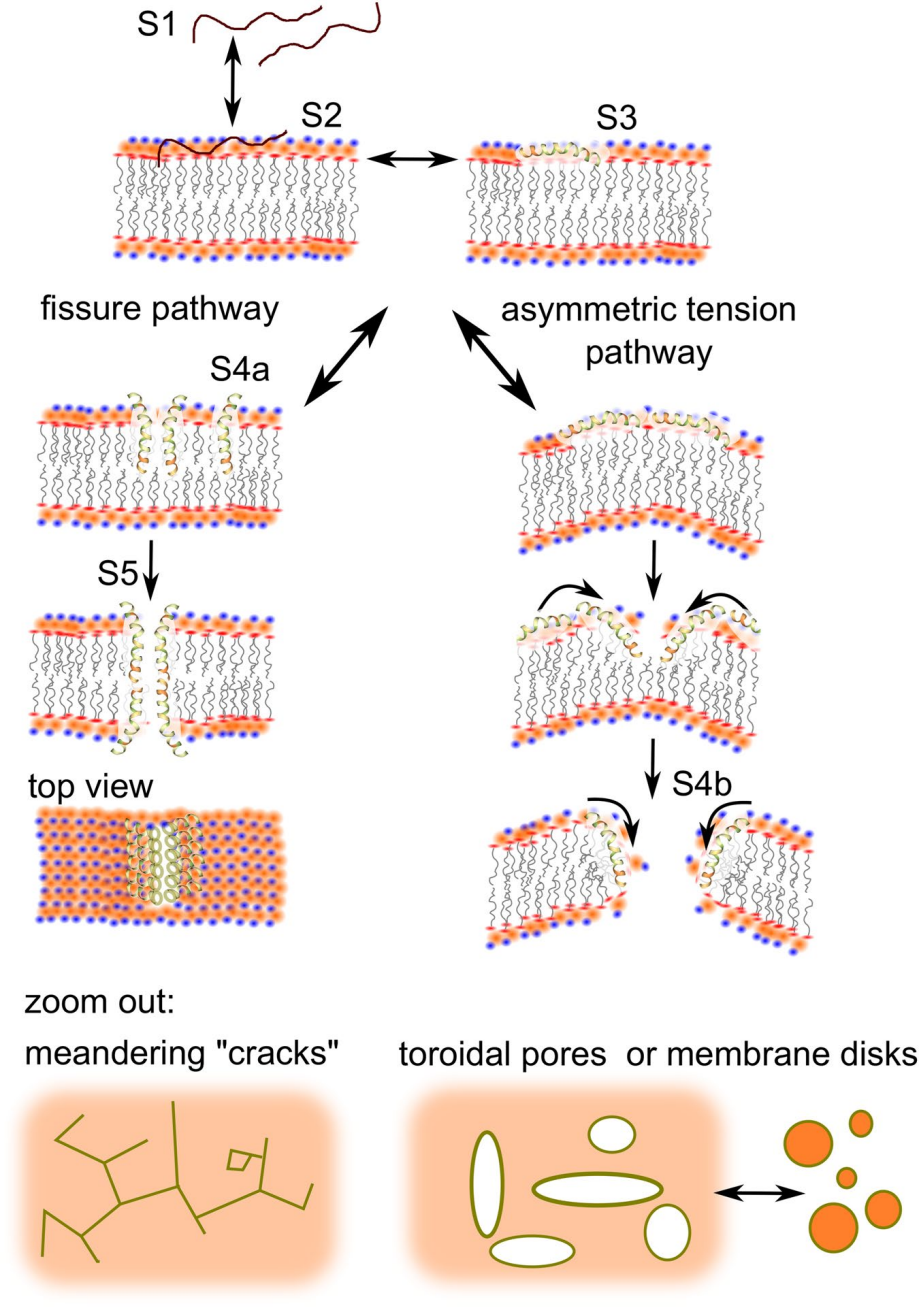
Hypothetic pathways of melittin pore forming mechanism. In the schematic representation of the lipid headgroups, red are ester, orange are phosphate and blue are choline moieties. The peptides are shown fully helical according to melittin crystal structure; it is likely that under physiological conditions the C-terminal segment is unstructured and in that form it directly associates to the lipid headgroups.

In terms of the mechanism, first the peptide has to adsorb to the membrane surface; this is a reversible dynamic process, yielding an equilibrium between the solvated (Fig. 4S1) and adsorbed (Fig. 4S2) states. The adsorbed peptide then folds into a helix (Fig. 4S3), in equilibrium with the unstructured S2 state. The folded peptide penetrates the membrane (Fig. 4S4a). Potentially, folding and penetration happen simultaneously, hence it is unclear whether S3 is a distinct state.

It should be assessed whether monomeric or aggregated forms are more likely to penetrate the membrane. The argument for the latter is that aggregation of amphiphiles is energetically favoured and it may promote membrane insertion by obscuring hydrophilic regions and exposing hydrophobic areas that would reduce the energy of the insertion into the hydrophobic membrane core. Such bundling-and-insertion process was described for the barrel-stave pore forming alamethicin^94–96^. However, this is not consistent with a toroidal pore model where lipid molecules separate the peptides in the torus. Furthermore, if the peptides are still exposed to the aqueous environment on the membrane surface at the time of aggregation, it is more likely that the aggregation would be driven by hydrophobic interaction, exposing the hydrophilic residues, and thus inhibiting insertion instead of promoting it. Hence, the membrane insertion is assumed to be in monomeric form in this pathway.

The peptides may also aggregate after insertion. Considering that the polar residues are distributed over ~23%^97^ of the helix circumference, a dimeric, or even a circular aggregate would not be stable (i.e. melittin is unlikely to form a barrel-stave pore); however a linear aggregate is possible (Fig. 4S5). The formation of such linear aggregates may allow increased water penetration along these “cracks”.

Considering the shallow penetration of the peptide into the membrane, in the absence of a lipid torus it cannot open a pore, unless there is a pathway for the peptides to transfer to the inner leaflet. Direct transfer is energetically prohibited due to the high charge of the hydrophilic terminus. However it is possible that crack formation as per above would reduce the energy barrier and allow peptide flip-flopping to the inner leaflet, likely through density fluctuations (transient pores) along the crack. That way the cracks can potentially open into fissures, stabilized by arrays of peptides embedded in the opposite leaflets. There is microscopy evidence of the formation of such fissures^54^. Importantly, this fissure model does not necessitate the formation of a toroidal structure, i.e no need for lipid interdigitation between the peptides. The peptide transfer to the inner leaflet does not represent a different energetic state.

### Asymmetric tension hypothesis

The formation of toroidal pores requires a different pathway. The peptide has to alter the membrane curvature, hence it has to remain associated with the headgroup zone (Fig. 4 right). This is possible in the presence of phosphatidylglycerol lipids that allow deeper water penetration into the headgroup zone due to the smaller size than the choline group of the neutral lipids, and the ability of water to hydrogen bond to the alcohol moieties of the glycerol. Water can thus access the side chains of the polar residues of the N-terminal helix of melittin and restrict their ability to “sink” into the hydrophobic membrane core. These side chains may also bind directly to the phosphate or ester oxygens of the lipids, as mentioned above. Thus melittin would act on the surface, remain embedded at the boundary between the apolar core and the headgroup zone, and, due to its size, apply lateral pressure to the outer leaflet. Continuous adhesion of peptides would introduce an increasing difference in the surface tension between the two membrane leaflets. When the difference reaches a threshold, the membrane would break, with a high likelihood that the peptides are carried into the breach, forming a toroidal pore; this is a distinct energetic state (Fig. 4S4b).

### Interpretation of melittin action as a function of membrane composition

It is clear from the experimental data that melittin interaction, and hence the disruption pathway is a function of membrane composition. A key outcome of the experiments is that in domain separated membranes DMPC:cholesterol 9:1 and DMPC:DMPG 4:1 melittin interacts preferentially with one of the domains. Due to the similarity of the fingerprints to the neat DMPC case, melittin appears to preference the DMPC rich domain. Hence, in a competitive environment, the membrane-inserted state is the lower free energy state for melittin. According to the models outlined above, melittin can follow the fissure pathway in these cases, as in neat DMPC, including up to 4 membrane bound states (unstructured; structured on the surface; membrane penetrated; aggregated). The analysis of the f-D curves suggests that the first stage is a composite of the individually opposing dissipation trends of the population of the two surface bound and the membrane penetrated states, whereas the second stage is the result of crack formation. The membrane remains mostly intact.

It is also clear that DMPC:DMPG (3:2) case shows melittin as a surface acting peptide that breaches the membrane once a certain surface concentration is reached; it follows the asymmetric tension pathway through the surface bound unstructured, surface bound structured and torus bound states (the latter one would differ in free energy due to the altered local membrane curvature). The first two, as in case of the fissure pathway, cannot be distinguished from the viscoelastic fingerprints, yielding the first stage of the mechanism; the second stage is consistent with pore opening. The third stage is thus the collapse of the membrane back to the sensor surface as seen, and explained, with other surface acting peptides^64,70^.

DLS results confirmed the dissolution of the membrane into uniform sized mixed micelles. While literature normally classifies the peptides into pore formers and carpet-like membrane disruptors, it is easy to see that the mechanism can switch between the two as a function of peptide concentration. The edge of the “toroidal pore” can have convex or concave curvatures; structurally and energetically it is not much different from a peptide-stabilized membrane nanodisc (Fig. 4 bottom right). Therefore, it is highly likely that high concentrations of the peptide can invert the pore structure into a series of islands. The same is true for the fissures described above. Hence at high peptide concentration melittin may dissolve the membrane; cracks in a melittin-exposed DMPC:DMPG (4:1) membrane have been imaged before^54^.

Melittin action was much attenuated and slightly altered in unsaturated or high cholesterol content (8:2) membranes compared to neat DMPC. It is likely that this is a simple effect of a physical obstruction. In both cases the membrane core is less accessible to the peptide hence the membrane inserted monomeric state has a higher free energy than in neat saturated lipids, shifting the equilibrium towards the surface bound state. Co-existence of the two states explains the brief [-f, 0] trendline. The second stage of the QCM fingerprints is thus the result of retaining a substantial population of peptides in the headgroup zone and concomitant modulation of membrane tension (blistering), but dampened by the presence of a population of membrane inserted peptides, explaining the observed limitation of the dissipative processes. The final stage indicates the collapsed membrane as discussed before.

## Conclusion

The action of melittin on different lipids and mixtures was characterized by using dye leakage and QCM fingerprinting measurements. The results demonstrate that melittin is able to breach all type of membranes: neutral and charged, made of saturated or unsaturated lipids, and irrespective of whether the membranes contained cholesterol. The QCM results revealed melittin-membrane interaction is a multi-step process, and that melittin can follow different disrupting pathways in different membranes, switching between a surface acting and an inserting mode. The main controls of the mode of action were identified as the presence or absence of phosphatidylglycerol and the structure of the membrane core. It was noted that in domain separated membranes melittin shows a preference for the DMPC-rich domain. The data allowed the construction of two hypothetic disruption models: the fissure-forming model describing a membrane penetrating pathway and the asymmetric tension model leading to toroidal pores through surface action.

## Supplementary information

Supplementary Dataset 1
