# Supplementary material for: Nano-viscosimetry analysis of the membrane disrupting action of the bee venom peptide melittin
Source: Sci Rep. 2019 Jul 25;9:10841. doi: 10.1038/s41598-019-47325-y (PMC6658469; doi:10.1038/s41598-019-47325-y)
Supplement: Supplementary file 1 — Supplementary Dataset 1 [file 41598_2019_47325_MOESM1_ESM.docx]

**Supplementary Information**

Nano-viscosimetry analysis of the membrane disrupting action of the bee venom peptide melittin

Sara Pandidan and Adam Mechler

*La Trobe Institute for Molecular Science, La Trobe University, Melbourne, Australia*

**Dynamic Light Scattering (DLS) experiments**


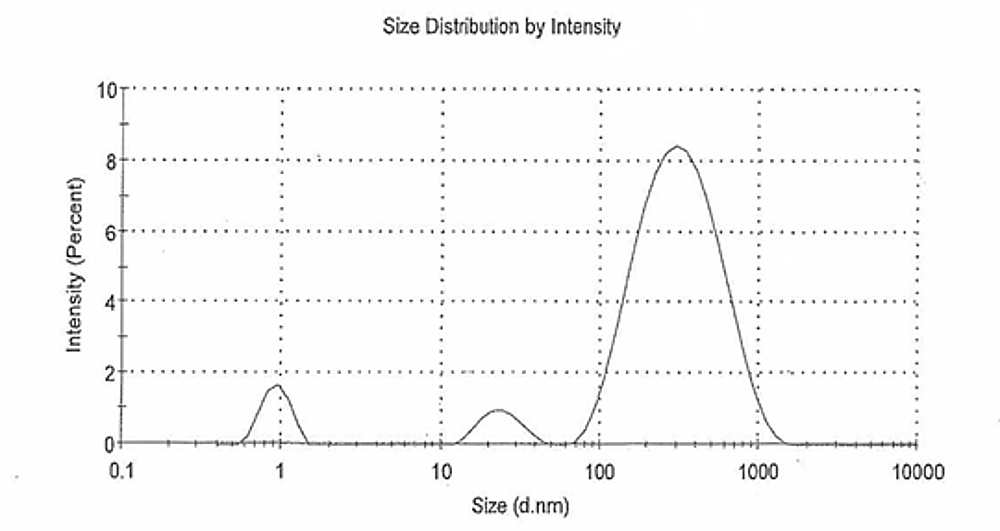


Figure S1. Representative DLS size distribution graph of the product of melittin membrane disruption at high peptide concentration.
